# Should Dairy Cattle Be Trained to a Virtual Fence System as Individuals or in Groups?

**DOI:** 10.3390/ani10101767

**Published:** 2020-09-29

**Authors:** Patricia I. Colusso, Cameron E. F. Clark, Sabrina Lomax

**Affiliations:** School of Life and Environmental Sciences, Faculty of Science, The University of Sydney, Camden 2570, NSW, Australia; cameron.clark@sydney.edu.au (C.E.F.C.); sabrina.lomax@sydney.edu.au (S.L.)

**Keywords:** virtual fence (VF), audio tone (AT), electrical pulse (EP), VF stimuli, treatment, individual, group, training phase, crossover phase, positive-punishment associative learning

## Abstract

**Simple Summary:**

A virtual fence (VF) system is being evaluated for commercial implementation in the Australian livestock industries. For this to work in dairy systems, cows will require training to learn the association between paired stimuli for livestock containment. We aimed to understand if cow learning and response to VF stimuli would differ when trained as individuals or in groups in a controlled experimental environment. Twenty-three dairy cows were trained to a VF as individuals or in groups of 5–6, and then moved to the alternate context to test the retention of learning. Cows trained in groups were more likely to interact with the VF when tested as individuals, indicating they might rely on the response of their conspecifics rather than directly receiving stimuli themselves. It is important that all individuals learn the association between stimuli to ensure they remain within a boundary, and to minimise potential welfare implications on animals that do not learn. However, training individual cattle is impractical, therefore, further work should evaluate effective group training protocols that provide the time and space for all individuals to learn the VF.

**Abstract:**

Pre-commercial virtual fence (VF) neckbands (eShepherd^®^, Agersens, Melbourne, Vic, Australia) can contain cows within a designated area without the need for physical fencing, through associative learning of a paired audio tone and electrical pulse. Cattle are gregarious, so there may be an impact of herd mates on the learning process. To evaluate this, a VF was set 30 m down one of three test paddocks with a feed attractant 70 m past the VF. Twenty-three Holstein-Friesian cows were all fitted with VF neckbands and trained as individuals or in groups (5–6) for four 10 min tests; then, cows were crossed over to the alternate context for two more 10 min tests. The number of cows breaking through the VF and the number of paired stimuli reduced across time (from 82% to 26% and 45% to 14%, respectively, *p* < 0.01). Cows trained in a group (88%) were more likely to interact with the VF in the crossover compared to those trained as individuals (36%) (*p* < 0.01), indicating an influence of group members on individual cow response. Individual training is impractical, therefore, future research should evaluate group training protocols ensuring all cows learn the VF to avoid any adverse impacts on animal welfare.

## 1. Introduction

In Australia and New Zealand, pasture-based dairy systems rely on the accurate allocation of pasture for profitability [1], enabled by a mix of permanent and manually shifted temporary fencing. A potential alternative to manually shifting fencing is the use of virtual fence (VF) technology, which could provide a greater level of flexibility and real-time, remote control over livestock and pasture allocations [2,3,4].

Virtual fencing systems rely on operant conditioning, positive punishment associative learning between a conditioned stimulus (an audio tone, AT) and unconditioned stimulus (an electrical pulse, EP) [5] administered to an animal via neck-mounted devices [6,7]. Positive punishment associative learning training method utilises a negative/aversive stimulus to alter an animals behaviour to avoid receiving the stimuli [8]. The VF neckbands use GPS positioning to dictate the VF boundary. The area within the VF boundary is referred to as the inclusion zone, in which the animals are free to move around without receiving stimuli. The area outside of the VF boundary is referred to as the exclusion zone, which is the area in which the animals are being excluded from and where stimuli will be delivered. Stimuli delivery is based on both locomotion and direction an animal is facing, regarding the inclusion zone. The AT is delivered to an animal when it approaches a VF boundary; if the animals continue forward and breaks through the VF, an EP is administered. If the animal stops movement and/or turns back towards the inclusion zone, all stimuli will cease. Once the association has been established, the animal should respond to the AT to avoid the EP and remain within the VF boundary. Positive punishment training methods can have welfare implications, particularly if delivery is inconsistent, which affects an animal’s ability to learn [8,9]. Previous studies have demonstrated that the stress response to handling was greater than the response to VF stimuli in sheep [10], and the response of cattle to electrical stimuli was comparable to an electric fence [11]. The consistent delivery of stimuli (AT before EP) based on the animal’s behaviour, location and direction ensure predictability and controllability [12,13,14]. However, individual variation in learning rate and the response has been identified in the VF literature [13], which may impact on welfare and the effectiveness of this technology in animal containment, which requires further study. 

Multiple studies in cattle using different versions of a pre-commercial prototype (eShepherd^®^, Agersens, Melbourne, Vic, Australia) VF device have reported on the impact of age and breed [15], moving VF fences [16], containment from riparian areas [17], spatial utilisation [7], and animal behaviour and welfare [5,11,13,15,18]. Findings suggest that cattle can learn a VF with greater than six interactions [13], as individuals [5,6,15,18] and in groups of up to 12 animals [7,16,17]. The studies have all identified variation between animals in the learning of and response to a VF, however, there is a paucity of data on the impact of VF training or training context.

Cattle can be trained using classical and operant conditioning techniques, but as a gregarious species, they also learn through social facilitation [19,20]. The herd setting provides individual animals in differing ways in which they can learn from their conspecifics. Observational learning is common, which involves animals visually observing conspecifics and responding, rather than through direct experience [21]. Across species, individuals have been shown to imitate the behaviour of conspecifics, regarding movement patterns [22], foraging methods [23] and even social interactions [19,23,24]. In nature, social facilitation has benefits for survival (such as learning) to avoid danger (such as from predation or, consuming toxic flora [24]), and for cattle, this can be learning how to graze [25] and locating resources [26,27]. Social facilitation is beneficial for cattle learning, however, associative learning requires individuals to have direct exposure to paired stimuli. Therefore, the presence of herd mates during the learning process may impact on an individual’s ability or time required to directly interact with the VF stimuli and associatively learn. 

Animals trained using associative learning can link sensory cues (visual, olfactory and auditory) [28,29,30,31]. If these cues are altered, the ability of an animal to learn the desired response to a stimulus may be impacted [29,31]. This is important to address as cattle in both intensive and extensive systems experience constant changes in spatial and social settings as a component of management. Typically, dairy cattle are managed separately within age groups and milking cycles [32] and can be at differing locations as cows move in and out of herds as they dry off. The frequent changes in herd compositions and location of dairy cattle is an important factor to consider when training them for containment with a virtual fence. Identifying learning requirements and appropriate training methods for cattle will not only be necessary for the successful application of this technology into industry, but will also be vital for acceptable animal welfare.

There has been no published work evaluating the impact of training contexts or the effect of conspecifics on individual cow learning of and response to VF stimuli which is required to better understand and facilitate optimal cattle learning across herd contexts. Therefore, this experiment aimed to compare how individual dairy cows learn a VF when trained on their own or in a group, and to determine how this learning was retained across social contexts. 

## 2. Materials and Methods 

This experiment was conducted at The University of Sydney’s research farm “Mayfarm” near Camden, NSW, Australia, between June and July 2018. All experimental protocols were approved by the University of Sydney’s Animal Ethics Committee (Project 2018/1306).

### 2.1. Experimental Design 

Twenty-three Holstein-Friesian dry cows (Mean age of 6 years old, min: 4 years and max: 9 years; mean weight of 641 kg liveweight, min: 506 kg and max: 777 kg liveweight) were used in the experiment. Cows were allocated a DR (as described below) and ordered from high to low ranked cows into an Excel^®^ file (version 1908, 2016). The cows were then randomly assigned a number using the RAND function in Excel^®^ (Microsoft Corporation, Redmond, WA, USA) and sorted by descending numerals before being split in half to form two groups (n = 1–12). This ensured an equal allocation of dominant and submissive individuals within the treatment groups. A crossover latin square design was used to evaluate learning and response within and across treatments. Two treatments were implemented: (1) Individual exposure to a VF; and (2) Group (n = 5–6 cows per group) exposure to a VF. The experiment was conducted across two phases: (1) An initial training phase in which the animals were trained to VF stimuli within their allocated treatment; and (2) a crossover phase to evaluate how they retained the learning when moved to the alternate treatment. For example, the cows that were trained as individuals in the training phase were then tested in groups in the crossover phase and vice versa. The cows in the group treatment were continually randomised (RAND function in Excel^®^) into subgroups before testing. This was done to ensure the group formations provided an accurate representation of differing herd dynamics in traditional herds. There were a total of twelve group combinations across all six tests.

The VF pre-commercial prototypes (eShepherd™, Agersens Pty Ltd., Melbourne, VIC, Australia) have been previously described [7,17]. Briefly, the VF experimental prototype neckbands consisted of a strap with a counterweight (total weight approximately 1.4 kg) and an electronic unit (approximately 725 g and 17 cm L × 12 cm W × 13 cm H) positioned on the top of each animal’s neck, with two electrodes that contact the skin on the right side of the neck. Using GPS technology, the unit monitored cow movement and provided real-time data on cow location, heading and speed. A VF boundary separating the inclusion from the exclusion zones, specified using GPS coordinates, was transmitted to the unit using a radio frequency link. As the animal approached the VF boundary, the unit emitted a distinctive AT within the cow’s hearing range. If the cow remained at that location or turned away, no EP was applied. If the animal continued to move through the VF boundary into the exclusion zone, the unit delivered a short, sharp pulse in the kilovolt range (values are commercial in confidence). This sequence of an AT followed by the EP was repeated if the animal continued through the VF and into the “exclusion zone”. The date, time, GPS location and “event” which included where the cow was located, concerning the inclusion zone and details of stimuli delivery, were logged for later download from the unit.

**Figure 1:**
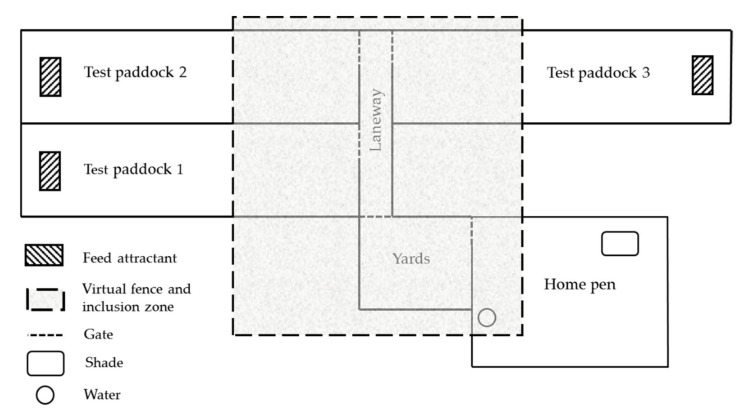
Diagrammatic representation of the experimental site. Figure not to scale. The experimental site consisted of a home pen to house animals outside of testing. Each day of testing a virtual fence (VF)was activated (shaded area) and cows were moved from the home pen into the holding yards which were contained within the VF inclusion zone. For each test, cows were moved either as individuals or in groups along a laneway and through the access gate into the test paddock. Testing was randomly alternated between three test paddocks across tests. Test paddocks 100 × 20 m contained a feed attractant of Lucerne cubes at the end (70 m from VF) and were mowed so that cows could not graze pasture during the test. The VF was positioned 30 m from the start of each paddock.

All cattle were naïve to the VF neckbands before the experiment. Every cow was habituated to inactive neckbands for one week before the experimental period. Cows were monitored daily, with no adverse responses observed. On day one, the cows were weighed (Gallagher G02601 TWI weigh scale, Gallagher Group Ltd., Hamilton, New Zealand), identified with numbers on each flank for ease of identification (Tell tail, GEA Fil Ltd., Mount Maunganui, New Zealand) and a VF neckband was assigned and fitted in a cattle crush. Neckbands required attachment in a cattle crush as per commercial recommendations and for both cow and human safety. Once the neckbands were fitted, the cattle were released into the home pen for two days to habituate (Figure 1). In the home pen, cows were offered a ration of Lucerne hay (dry matter 89%, crude protein 16.4%, metabolisable energy 8.5 MJ ME/kg DM), which was offered at a maintenance level determined as [33]:

MJ ME = 0.67 × BW^0.75^ (1)

Three test paddocks (100 m × 20 m) (Figure 1) were used to evaluate cattle training methods in a controlled environment for all six tests. A feed attractant of 2 kg of lucerne cubes per cow (Multicube; dry matter 88%, crude protein 18%, metabolisable energy 9.1 MJ/kg DM, Yarrawonga, Vic, Australia) was placed at the end of each test paddock outside the VF as depicted in Figure 1. The paddock design was to emulate a strip grazing scenario in an artificial controllable context. The feed attractant was used as a representation of the next day’s pasture allocation; thus, animal containment from a feed source could be evaluated.

On days three to seven, the cattle were allowed to access the feed attractant at the end of each of the three test paddocks (Figure 1). Cows were provided access to each test paddock with the feed attractant in their treatment groups between 0800–1100 h and again between 1300–1600 h each day for two days, alternating between test paddocks each time. This continued until all cows walked unassisted to the feed. Following this, cows were then provided access to the test paddocks individually for two more days following the same method above.

### 2.2. Determining Social Order 

Agonistic behaviours that occurred between pairs of cows were recorded via video (Sony HDR-AS300 action cam, Tokyo, Japan) and live observations were used to determine dominance rank (DR). Live observations were opportunistic recordings of interactions. This was initially completed in the habituation period to balance the division of cows into the two treatment groups. After which, live observations were continually recorded between and during tests. Cows were held in the cattle yards (21 m × 17 m) without feed for two hours, and two video cameras were set up in the yards to capture the agonistic behaviours without a human observer. This was completed three times during the habituation period. Only agonistic behaviours that had a clear winner (dominant individual) and loser (subordinate individual) were used to assign a “win” or “loss” score for each cow. The main agonistic behaviours included were approaches that involved one cow approaching another to move them, head bunts or pushes [34]. These agonistic behaviours were only recorded if it resulted in the receiving cow avoiding and/or retreating from the aggressor [35]. All recordings from the video footage and live observations were collated into an Excel^®^ file (version 1908, 2016) and a DR was calculated for each cow using the following formula [36]:

DR = (Ai − Bi)/N(2)

A = number of wins

B = number of losses 

N = total number of interactions (Ai + Bi)

Each cow was allocated a DR of between −1 to +1 with the greater values relating to dominance and lower values to subordinance [36]. A total of 200 agonistic observations were recorded throughout the experiment. 

### 2.3. Virtual Fence Testing 

Four tests were conducted in the training phase, and a further two tests were conducted in the crossover phase, with one test being conducted per day. Testing continued until cows no longer walked toward the feed attractant voluntarily, which occurred after two tests in the crossover phase, resulting in the conclusion of the experiment. 

On each day of VF testing, the cows were moved from the home pen to the cattle yards and divided into their treatment groups based on their number identifiers. A test paddock was then randomly selected each morning by selecting a number out of a bag (1–3) to ensure all paddocks were used and to reduce the impact of spatial memory and/or avoidance associated with the EP, and to allow for the transfer of learning between locations [16,37,38]. 

The VF neckbands were activated between 1000 h–1400 h up to 30 min before testing. During this time, cows were held in the yard, which was enclosed within the inclusion zone (Figure 1). The online user interface was used to create the VF boundary and inclusion zone. The exclusion zone represented everything outside the inclusion zone, where VF stimuli would be delivered. The VF was set 30 m from the entry to the paddock (70 m from feed attractant) (Figure 1) to provide adequate space between the VF line and the feed attractant, and to allow sufficient time for the animals to receive and respond to VF stimuli. Before a test was conducted the cow(s) were moved from the yards, along the laneway and into the test paddock. Once the cow(s) entered the test paddock, it was closed off, and the test began. The cows were left in the test paddock for a total of 10 min to interact with the VF and or attempt to approach the feed attractant (Figure 1). No external pushing or mustering were applied to the cow(s) during this time. After 10 min, the test ended, and an observer would enter the test paddock and walk the cow(s) back to the neutral holding zone (yards). Once testing was concluded for the day, the VF was deactivated, and the cows were returned to the home pen. 

Between 0700 h–0900 h each day all cows were allowed to access the feed attractant in the selected laneway, within their groups, without receiving stimuli, to increase the likelihood that they would continue to participate in subsequent tests [18]. This positive reinforcement was also used to prevent spatial associations with the paddocks to evaluate the cow’s learning to VF stimuli rather than spatial locations.

### 2.4. Data Collation and Processing

The VF neckbands logged a GPS location and stimuli details (delivery of stimuli or not) at an alternating frequency (between seconds and minutes), dependent on the animal’s movement and/or location relevant to the VF. Date and time-stamped GPS location, and stimuli details (AT and EP) were downloaded from the neckbands. The neckbands were removed from the cows in the cattle crush to retrieve the SD card and download data after the fourth test and again at the conclusion of the study. The logged txt files were downloaded from neckbands to produce an Excel^®^ file (version 1908, 2016). Stimuli data was filtered from the Excel^®^ file and stacked for each cow by training and crossover phase. The stacked data was categorised by stimuli and collated into a total count of AT and EP for each animal per phase. The proportion of paired stimuli was determined by dividing the EP by the AT count for each animal. There were a total of 238 AT and 88 EP across all six tests not included in the stimuli analysis. This stimulus was delivered when the cows had reached the feed and were eating. As the stimuli were delivered during eating, it was not used in the analysis of VF learning and response. One audio tone was incorrectly delivered outside the test period in the cattle yard and was removed from the analysis. It was calculated that for 23 animals over six tests, there should have been 138 VF complete data records (VF data log containing GPS and stimuli data per cow). However, neckband technical issues resulted in only 51 complete data records being retrieved. There was a total of 34 complete data points for groups and 31 for individuals in phase 1, and 9 for groups and 11 for individuals in phase 2. The missing and incomplete recordings resulted in uneven VF stimuli data between phases and treatments, limiting detailed stimuli analysis. Visual observations of cows receiving stimuli, reaching the feed attractant, and returning to the inclusion zone were recorded as binomial data (yes = 1, no = 0). Receiving VF stimuli were determined if an animal interacted with the VF, based on the known location of the VF (mapped out prior to every test with a non-test neckband). If an animal continued to break through the VF and reach within 10 m of the feed attractant and/or reached the feed and began eating, it was recorded as the animal reaching the feed. An animal could return to the inclusion zone after they received stimuli and/or reached the feed. Therefore, an animal that reached the feed and/or returned to the inclusion zone had to have first interacted with the VF and received stimuli.

Three video cameras (Sony HDR-AS300 action cam) were used to record individual cow’s behavioural responses to VF stimuli. One observer reviewed the timestamped footage (HH:MM:SS) for each animal and matched available VF neckband data to the corresponding behavioural response as per the ethogram shown in Table 1 [18]. The behaviours presented include only behavioural changes in response to VF stimuli. For example, if an animal’s behaviour remained the same before and after stimuli were delivered, it was not included. The behaviours in response to stimuli were then divided into two categories as correct or incorrect based on the directional change of the cow’s spatial position relative to the inclusion zone. A correct behavioural response referred to any behaviour that kept the animal within the inclusion zone (e.g., turn around), whereas an incorrect behavioural response referred to behaviours that kept the animal moving forward towards the exclusion zone (e.g., walk forward). A reactive response was included to represent behavioural changes that were neither correct nor incorrect (did not change the spatial position or direction of the animal), but merely a non-directional reaction to the VF stimuli (Table 1). All behaviours across both phases were combined, and the responsive behaviours at the start of the AT and EP were collated into a percentage of total behaviour (out of 100%) and frequency per cow (Table A2 and Table A3). Furthermore, the mean frequency of each behaviour was tabulated, including the standard deviation and range. Similar behaviours were combined, for example—the head position of the animal (head down and head up), and the type of forward locomotion (walk and trot forward). 

### 2.5. Statistical Analyses 

Statistical analyses were conducted in Rstudio© (v1.2.5019) [39], an integrated development environment for R (v 4.0.2) [40]. Live observational data were analysed using a series of Generalised Linear Mixed Models (GLMM) (family = binomial, link = logit) with lme4 package [41]. For the binomial data, the response variables used were ‘Receive VF stimuli’, ‘Reach feed’ and ‘Return to inclusion zone’ with Phase (training and crossover) and Treatment as the fixed effect, with Cow ID as a random effect. Within the training phase, two GLMMs were used for the response variables ‘Reach feed’ and ‘Return to inclusion zone’ with Test as the fixed effect and Cow ID as a random effect. 

Stimuli data were analysed using a third GLMM (family = negative binomial, link = log) with MASS package [42] for AT count and EP count analysing the fixed effects of Phase and Treatment, with Cow ID as a random effect.

All GLMM model residuals were graphically inspected using the DHARMa package [43], and overdispersion was checked using the dispersion function in lme4 [41]. 

The proportion of paired stimuli (EP:AT) data were log-transformed (e/0.125) for normal distribution. The residuals were graphically inspected for normality and heteroscedasticity using DHARMa package [43] and dispersion function in lme4 [41]. One linear mixed model (LMM) was used to analyse the proportion of paired stimuli, with the fixed effects of Phase and Treatment, and Cow ID as the random effect.

All models were refined to contain only significant fixed effects; however, treatment remained in the final models as a controlling factor, as well as for reporting (Table A1). This process involved comparing the multiple model variations (using the Akaike information criterion (AIC, [44]), chi square and *p*-values) [45] and selecting the best-fitted models before applying a likelihood ratio test (full models containing the fixed and random effects, compared to the null model containing only the random effect) [46]. Post-hoc analyses were conducted using the emmeans package [47] for significant full models, with results presented on the back-transformed scale (estimated marginal means ± standard error). Behavioural responses to stimuli were tabulated and presented as a percentage of overall behaviour (out of 100%) and frequency for each cow across both phases, and further summarised by mean percentage of each behaviour, including the standard deviation (SD) and range (minimum and maximum). 

An alpha of *p* < 0.05 was set to determine the level of significance and a statistical tendency at 0.05 < *p* < 0.1.

## 3. Results

There was an interaction between phase (Training and crossover phase) and treatment for cows receiving VF stimuli (Table 2). Cows had an increased probability (88%) of receiving VF stimuli as individuals, regardless of phase (Table 2). Cows trained as individuals had a lower probability (36%) of receiving VF stimuli tested as a group in the crossover phase. The cows trained in groups were more likely (88%) to receive VF stimuli when tested as individuals in the crossover phase (88%).

Cows were more likely to cross the VF and reach the feed attractant during the training phase (82%), as compared to the crossover phase (26%) (Table 3). Within the training phase, there was a statistical tendency in the reduction of cows reaching the feed by test 4 (21%) as compared to test 1 (98%, Table 4). 

Cows were more likely to return to the inclusion zone after receiving VF stimuli in the crossover phase as compared to the training phase (Table 5). Within the training phase, there was an increase in cows returning to the inclusion zone, with 100% of cows returning by Test 3 and 4 (Table 4). 

There was a reduction in the number of VF stimuli delivered between phases, with cows receiving more stimuli during the training phase as compared to the crossover phase (Table 6). The proportion of paired stimuli (EP:AT) reduced between phases, with 45% of VF stimuli being paired during training compared with only 14% during the crossover phase (Table 6).

The behavioural response to the AT and EP varied between individual cows (Table A2 and Table A3), and within behaviours, as seen by the range in frequency displayed (minimum and maximum, Table 7).

Commonly observed behaviours were also identified (Table A2 and Table A3) and explored further in Table 7. The most frequently displayed behaviour at the start of the AT with 32 observations was walking forward, followed by 28 observations of head down/up, 25 observations of stopped locomotion and 19 observations of turning away (Table 7). The most frequent behaviour at the start of the EP was reflex with 38 observations, 17 observations of turning away and 16 observations of trot/running forward (Table 7). 

## 4. Discussion

This experiment reveals the response of twenty-three dairy cattle to VF stimuli within different grouping contexts across time. It is the first work to compare dairy cow learning and response to VF stimuli when tested as an individual or within a group and evaluation of how learning is transferred between these contexts. Most cattle responded correctly to VF stimuli by test 4. This is evident by the reduction in cows breaking through the VF to reach the feed, with all the cows returning to the inclusion zone by the end of the training period, indicating learning of the VF cues. Previous studies have reported similar results, with cows increasingly returning to an inclusion zone after interacting with a VF within three and four test sessions [5,15]. In the current experiment, cow learning was evident by the reduction in paired stimuli over time, which demonstrates the cattle’s ability to associate the AT with the EP, and thus, alter their behaviour to avoid the EP. By test 5, the cows were responding in majority to the AT alone. Similarly, cows have been reported to respond to the AT alone after more than six interactions with the VF [18], between three [15] and four test sessions [18] as individuals, and within two [16] and four days [7] as groups on pasture. Due to the consistent delivery of an AT before an EP, the cows could predict, and thus, control their response to avoid the EP and remain within the inclusion zone [13]. The constraints of missing data have limited further analyses, regardless in combination with the live observations, we highlight the ability of the dairy cows to learn and respond to VF stimuli.

Training cows as a group may affect their ability to associate paired stimuli, and thus, may increase the time it takes for an individual cow to learn a VF. The cows trained in a group were 88% more likely to receive stimuli when tested as individuals, as compared to only 36% of cows trained as individuals. When trained on their own, cows had a greater opportunity to directly interact with the VF and receive paired stimuli. Anecdotally, the AT was loud enough to be heard by the human observers within 10 m and may have influenced cows responding to conspecific stimuli and/or the behaviour of their conspecifics in response to stimuli. For example, a cow at the front of the group received an AT and responded correctly by turning around, which triggered the surrounding cows to imitate her response without having directly experienced the stimuli. However, these indirect responses were not empirically measured. As a gregarious species, social facilitation plays a major role in cattle learning [22]. Individuals within a herd can learn through observations of conspecifics and adjust their own behaviour [23]. Social facilitation and herd response have been observed in cattle when learning or coming into contact with electrical stimuli [48,49]. A study focusing on training cattle to electric fences observed that one animal’s response to receiving an electrical shock resulted in the whole herd retreating from the electric fence location [49]. Similarly, social avoidance has been reported in beef cattle being excluded from a riparian zone by a VF, where one individual’s aversive response to the EP resulted in the whole herd turning away [17]. Further research is required to assess the role of social facilitation on cattle learning of a VF, as well as the perception of VF stimuli by neighbouring animals. 

Animal behaviour is a tool for understanding how animals are coping with their external environment and potential stressors [50]. Although missing data limited comparison of response to stimuli between individuals and across time, we identified a wide range of behavioural responses in the cows analysed, which aligns with previous studies [5,18]. A correct behavioural response should keep a cow within an inclusion zone, including turning away from or stopping in response to stimuli. We identified a high frequency of cows turning away from the AT and EP. Additionally, at the AT, cows would also stop movement, and turn back towards the inclusion zone at the EP. This aligns with the reduction in cows reaching the feed across time, from 82% in the training phase to 26% in the crossover phase, and the subsequent increase in animals returning to the inclusion zone from 48% to 84% in crossover phase. However, there was a high frequency of incorrect behaviours observed, where cows would continue walking forward, or trot or run into the exclusion zone following stimuli delivery. Similar incorrect responses have been identified in previous VF experiments as part of the learning process, in which these responses reduce over time as animals learn the stimuli association [5]. We observed avoidance type behaviours, including raising, lowering, shaking and/or turning of the head in response to the AT, and full body reflex in response to the EP’s. These behaviours may be an attempt to alleviate or avoid the stimuli [6,31], which were delivered via electrodes positioned on the right side of the animal’s neck. Variation in individual sensitivity to electrical stimuli and the accompanying behavioural responses [18,51], may comprise part of a stress response [50], though physiological measures of stress were not evaluated in the current study. There are also potential welfare implications if animals are unable to learn to associate VF stimuli. In this study, we did not observe any adverse behavioural responses; however, due to missing stimuli data, further evaluation of stimuli delivery was limited. This warrants further study. Previous research has reported no significant behaviour or welfare impacts in beef cattle [11] or in sheep [10]. However, long term behaviour and welfare monitoring should be conducted to identify any animals that are unable to learn, and would, therefore, not be suited to the VF system [13].

In this study, we have identified the importance of individual cows directly receiving stimuli to ensure associative learning; however, it is impractical to train cows as individuals in a commercial setting. The cows were trained in 10min test sessions which may have been insufficient duration for all individuals within the group to experience, and therefore, establish the associative learning of stimuli. Previous VF studies have demonstrated that cattle learn in groups within 48–96 h on pasture [7,16]; however, these studies did not directly evaluate methods for training cattle to a VF, but identified a longer duration for cattle response in a herd setting. Therefore, future work is needed to evaluate training protocols with an emphasis on maximising learning and minimising the impact on cow welfare. The test paddocks with a feed attractant were used to evaluate the learning of a VF. While this enabled consistency between tests and greater control over experimental conditions, cows learned the spatial location of the VF in each test paddock, and therefore, no longer walked toward the feed attractant after six tests resulting in unbalanced test sessions, due to early conclusion of the experiment. Therefore, future work should evaluate learning in a pasture-based setting to ensure the translation of results to industry. 

## 5. Conclusions

In this article, we demonstrated the ability of dairy cows to learn and respond to a VF when trained as individuals or in groups. However, when training groups of animals, a longer training period over days is required to ensure all individuals have enough time to experience and associatively learn stimuli, and to enable the transfer of learning across differing herd contexts and environments. Further research is needed to assess the role of hunger, feed and social motivations on dairy cow learning and response to a VF. Additionally, the role of social facilitation on individual cow learning and how to utilise this for effective animal training should be identified. These future research areas will be critical for ensuring acceptable animal welfare within VF systems through the identification of limitations, appropriate management practices and optimisation of this technology for intensive grazing systems. 

## Figures and Tables

**Table 1:** Ethogram used to describe behavioural responses to the audio tone (AT) start and electrical pulse (EP) start. The type of behaviour can be either a correct and or incorrect response to the VF stimuli. A correct response refers to behaviour changes that keep the animal within or returning to the inclusion zone. An incorrect response refers to behavioural changes that keep the animal moving forward towards the exclusion zone. Furthermore, a reactive response has been included to present behavioural changes that a neither correct nor incorrect, but a behavioural reaction to the VF stimuli.

Behaviour	Description	Correct/Incorrect /Reactive Response
Reflex	Immediate pause in locomotion followed by a whole-body tense/startle (Flinch).	Reactive
Head down	Head lowered towards the ground (can include animal with head touching the ground).	Reactive
Head up	Head raised above normal holding position, can include animal throwing their head up.	Reactive
Head turn	Head turned to the left or right (can include lowered and raised head positions).	Reactive
Head shake	Head shake quickly from left to right (can include lowered and raised head positions).	Reactive
Turn away left	Animal turned away from the stimuli towards the left (can include 90 and 180 degree turns, dependent on animal position, as it refers to the animal turning away from the stimuli).	Correct
Turn away right	Animal turned away from the stimuli towards the right (can include 90 and 180 degree turns, dependent on animal position, as it refers to the animal turning away from the stimuli).	Correct
Turn back left	Animal turning to left, back toward the inclusion zone away from the stimuli (can include 90,180 and 360 degree turns, dependent on animal position, as it refers to the animal turning away from the stimuli and facing the inclusion zone).	Correct
Turn back right	Animal turning to the right, back toward the inclusion zone away from the stimuli (can include 90, 180 and 360 degree turns, dependent on animal position, as it refers to the animal turning away from the stimuli and facing the inclusion zone).	Correct
Stop	Animal stopping all locomotive movement (can include animals that have paused and or standing still).	Correct
Walk backwards	Animal is taking backwards steps (refers to animals that have stopped forward locomotion and started to back up still facing the previous direction (generally the exclusion zone)).	Correct
Walk back	Animal is walking back to the inclusion zone (refers to animals that have responded, facing the inclusion zone to return).	Correct
Trot back	Animal is trotting back to the inclusion zone (refers to animals that have responded, facing the inclusion zone to return).	Correct
Walk forward	Animal continued to walk forward through the stimuli towards the feed attractant into the exclusion zone.	Incorrect
Trot forward	Animal trotting forward through the stimuli towards the feed attractant into the exclusion zone.	Incorrect
Run forward	Animal running forward through the stimuli towards the feed attractant and into the exclusion zone (can include animals that have jumped forward 2–4 legs off the ground).	Incorrect

**Table 2:** The model interaction between Phase and Treatment predicted probability ± standard error of mean (SE), upper and lower confidence intervals (CI) and *p*-value of cows receiving VF stimuli. Cows were tested as individuals or groups in the training phase; then treatments were crossed over for the crossover phase, so that individuals were tested as groups and groups as individuals.

Training Context	Probability of Cows Receiving VF Stimuli (±SE)						*p*-Value
	Training Phase			Crossover Phase			
	Predicted Probability ± SE	Upper CI	Lower CI	Predicted Probability ± SE	Upper CI	Lower CI	
Individual	0.88 ± 0.056	0.96	0.69	0.36 ± 0.112	0.97	0.64	<0.01
Group	0.82 ± 0.067	0.92	0.64	0.88 ± 0.061	0.62	0.16	0.91
*p*-value	0.52			<0.01			

**Table 3:** The model predicted probability ± standard error of mean (SE), upper and lower confidence intervals (CI) and *p*-value of cows reaching the feed after receiving stimuli between Phase and Treatment. Cows were trained in the training phase; then treatments crossed over for the crossover phase.

Reach the Feed	Predicted Probability ± SE	Upper CI	Lower CI	*p*-Value
Training phase	0.82 ± 0.063	0.92	0.64	<0.01
Crossover phase	0.26 ± 0.114	0.57	0.08	
Individual	0.60 ± 0.097	0.78	0.37	0.57
Group	0.51 ± 0.133	0.77	0.24	

**Table 4:** The model predicted probability ± standard error of mean (SE), upper and lower confidence intervals (CI) and *p*-value of cows reaching feed and returning to the inclusion zone during the training phase (Test 1–4) after receiving stimuli. Probability with different superscripts are different, ^a,b^ indicates statistical tendency between tests (0.05), for reaching the feed, ^A–D^ indicates a significant difference between tests (<0.04), for returning to the inclusion zone.

Virtual Fence Test during Training Phase	Predicted Probability ± SE Reach the Feed	Upper CI Reach the Feed	Lower CI Reach the Feed	Predicted Probability ± SE Return to Inclusion Zone	Upper CI Return to Inclusion Zone	Lower C Return to Inclusion Zone
1	0.98 ^a^ ± 0.031	1.0	0.58	0.00 ^A^ ± 1.70 × 10^−11^	0.00	0.00
2	0.81 ^a,b^ ± 0.133	0.97	0.33	0.00 ^B^ ± 3.03 × 10^−5^	0.04	0.00
3	0.80 ^a,b^ ± 0.139	0.97	0.32	1.00 ^C^ ± 3.06 × 10^−5^	1.00	0.97
4	0.21 ^b^ ± 0.193	0.83	0.02	1.00 ^D^ ± 2.70 × 10^−11^	1.00	1.00

**Table 5:** The model predicted probability ± standard error of mean (SE), upper and lower confidence intervals (CI) and *p*-value of cows returning to the inclusion zone after receiving stimuli between Phase and Treatment. Cows were trained in the training phase; then treatments crossed over for the crossover phase.

Return to Inclusion Zone	Predicted Probability ± SE	Upper CI	Lower CI	*p*-Value
Training phase	0.48 ± 0.071	0.64	0.33	<0.01
Crossover phase	0.84 ± 0.078	0.95	0.59	
Individual	0.66 ± 0.082	0.82	0.47	0.67
Group	0.71 ± 0.093	0.87	0.47	

**Table 6:** The model mean count of the audio tone (AT) and electrical pulse (EP) ± standard error of mean (SE), upper and lower confidence intervals (CI) and *p*-value of cows between Phase and Treatment. The proportion of electrical pulse (EP) to audio tone (AT) ± standard error (SE) is presented as the proportion of paired stimuli (AT + EP) the cows received between Phase and Treatment.

Stimuli	Phase and Treatment	Mean Stimuli Count ± SE	Upper CI	Lower CI	*p*-Value
Audio tone count	Training phase	5 ± 0.5	6.10	4.03	<0.01
	Crossover phase	3 ± 0.5	4.18	1.79	
	Individual	4 ± 0.5	5.35	3.05	0.2
	Group	3 ± 0.5	4.65	2.41	
Electrical pulse count	Training phase	3 ± 0.3	3.60	2.01	<0.01
	Crossover phase	1 ± 0.3	1.71	0.46	
	Individual	2 ± 0.3	2.59	1.13	0.3
	Group	1 ± 0.3	2.28	0.84	
Proportion of EP:AT	Training phase	0.45 ± 0.058	0.61	0.33	<0.01
	Crossover phase	0.14 ± 0.046	0.27	0.05	
	Individual	0.26 ± 0.045	0.38	0.16	0.8
	Group	0.27 ± 0.060	0.44	0.15	

**Table 7:** Summary statistics of the frequency of behaviour of all animals in response to the delivery of audio tone (AT) and electrical pulse (EP) stimuli from the virtual fence (VF) neckbands. Presented are the mean frequency per behaviour to the AT and EP ± standard deviation (SD), the minimum and maximum from the mean. * indicates data not captured for that behaviour.

Behaviour	AT Frequency Mean	AT Min	AT Max	Total Frequency	EP Frequency Mean	EP Min	EP Max	Total Frequency
Stop	2 ± 0.9	1	4	25	1	1	1	7
Turn away left/right	2 ± 1.6	1	6	19	1 ± 0.7	1	3	17
Turn back left/right	1 ± 0.7	1	3	12	1 ± 0.5	1	2	9
Walk/trot back	2 ± 0.9	1	3	13	1 ± 0.6	1	2	4
Walk backwards	1 ± 0.5	1	2	5	1 ± 0.6	1	2	4
Walk forward	2 ± 1.8	1	7	32	1 ± 0.4	1	2	13
Trot/Run forward	1 ± 0.5	1	2	16	1 ± 0.9	1	4	16
Head down/up	2 ± 2.4	1	8	28	1 ± 0.7	1	3	11
Head turn/head shake	2 ± 1.3	1	5	20	1 ± 0.5	1	2	5
Reflex	*	*	*	*	3 ± 2.4	1	10	38

## Appendix A

**Table A1:** Final model fixed effects confidence interval (CI) and *p*-values.—represents the reference category.

Outcome	Fixed Effects	Category	CI Lower	CI Upper	*p*-Value
Receive VF stimuli	Phase:Treatment	Intercept	-	-	0.039
		Crossover phase:Individual	0.13	4.31	
Reach the feed	Phase	Training phase (reference category)	-	-	<0.001
		Crossover phase	−4.27	−1.28	
	Treatment	Group (reference category)	-	-	0.558
		Individual	−0.76	1.73	
Return to inclusion zone	Phase	Training phase (reference category)	-	-	0.001
		Crossover phase	0.61	3.01	
	Treatment	Group (reference category)	-	-	0.670
		Individual	−1.22	0.72	
Reach the feed in the Training phase	Test	Test 1 (reference category)	-	-	0.004
		Test 2	−5.17	−0.29	
		Test 3	−5.24	−0.34	
		Test 4	−11.23	−1.89	
	Treatment	Group (reference category)	-	-	0.476
		Individual	−4.08	1.62	
Return to inclusion zone in the Training phase	Test	Test 1 (reference category)	-	-	<0.001
		Test 2	6.70	23.16	
		Test 3	23.68	52.60	
		Test 4	32.53	73.09	
	Treatment	Group (reference category)	-	-	0.737
		Individual	−10.91	8.22	
Audio tone count (AT)	Phase	Training phase (reference category)	-	-	0.003
		Crossover phase	−1.00	−0.20	
	Treatment	Group (reference category)	-	-	0.252
		Individual	−0.14	0.50	
Electrical pulse count (EP)	Phase	Training phase (reference category)	-	-	<0.001
		Crossover phase	−1.73	−0.56	
	Treatment	Group (reference category)	-	-	0.348
		Individual	−0.24	0.65	
Proportion of paired stimuli (EP:AT)	Phase	Training phase (reference category)	-	-	<0.001
		Crossover phase	−1.15	−0.42	
	Treatment	Group (reference category)	-	-	0.778
		Individual	−0.36	0.28	

**Table A2:** The responsive behaviour of cows to the audio tone (AT) over the experimental period (Training and crossover phase). The percentage number represents the percentage of each cows’ behaviour displayed in each behaviour category (of its 100% behavioural repertoire), with the frequency of the behaviour displayed in parentheses. The grand total of all behaviours listed at the bottom represents the overall percentage that behaviour was displayed by all cows. Behaviours similar in nature were combined.

Cow ID	Total AT	Head Turn/Shake	Head Up/Down	Stop	Trot/Run Forward	Turn Away Left/Right	Turn Back Left/Right	Walk Backwards	Walk Forward	Walk/Trot Back	Total
1	12		8.33%(1)	25.00%(3)	16.67%(2)		25.00%(3)		16.67%(2)	8.33%(1)	100.00%
5	8	12.50%(1)		25.00%(2)	25.00%(2)	12.50%(1)			12.50%(1)	12.50%(1)	100.00%
6	5	20.00%(1)		40.00%(2)			20.00%(1)		20.00%(1)		100.00%
7	6	16.67%(1)			16.67%(1)	33.33%(2)			16.67%(1)	16.67%(1)	100.00%
8	16	18.75%(3)	18.75%(3)	12.50%(2)			6.25%(1)		43.75%(7)		100.00%
9	9	22.22%(2)			11.11%(1)		22.22%(2)	11.11%(1)	22.22%(2)	11.11%(1)	100.00%
10	25	8.00%(2)	32.00%(8)	8.00%(2)		24.00%(6)	4.00%(1)	4.00%(1)	16.00%(4)	4.00%(1)	100.00%
11	4		25.00%(1)		25.00%(1)					50.00%(2)	100.00%
12	2		50.00%(1)						50.00%(1)		100.00%
13	12	8.33%(1)	50.00%(6)	8.33%(1)	8.33%(1)	8.33%(1)		16.67%(2)			100.00%
14	1		100.00%(1)								100.00%
15	6	16.67%(1)	16.67%(1)	16.67%(1)	16.67%(1)				33.33%(2)		100.00%
16	2					50.00%(1)			50.00%(1)		100.00%
17	4	25.00%(1)	25.00%(1)		25.00%(1)		25.00%(1)				100.00%
18	8		12.50%(1)	25.00%(2)	12.50%(1)	25.00%(2)	12.50%(1)		12.50%(1)		100.00%
19	10		10.00%(1)	10.00%(1)	10.00%(1)	30.00%(3)			10.00%(1)	30.00%(3)	100.00%
20	1			100%(1)							100.00%
21	11	18.18%(2)		36.36%(4)					45.45%(5)		100.00%
22	14	35.71%(5)	21.43%(3)	14.29%(2)		7.14%(1)				21.43%(3)	100.00%
23	7			14.29%(1)	28.57%(2)	14.29%(1)	14.29%(1)	14.29%(1)	14.29%(1)		100.00%
24	7			14.29%(1)	28.57%(2)	14.29%(1)	14.29%(1)		28.57%(2)		100.00%
Grand Total	170	11.76%(20)	16.47%(28)	14.71%(25)	9.41%(16)	11.18% (19)	7.06%(12)	2.94% (5)	18.82%(32)	7.65%(13)	100.00%

**Table A3:** The responsive behaviour of cows to the electrical pulse (EP) over the experimental period (Training and crossover phase). The percentage number represents the percentage of each cows’ behaviour displayed in each behaviour category (of its 100% behavioural repertoire), with the frequency of the behaviour displayed in parentheses. The grand total of all behaviours listed at the bottom represents the overall percentage that behaviour was displayed by all cows. Behaviours similar in nature were combined.

Cow ID	Total EP	Flinch	Head Turn/Shake	Head Up/Down	Stop	Trot/Run Forward	Turn Away Left/Right	Turn Back Left/Right	Walk Back	Walk Backwards	Walk Forward	Total
1	5	20.00%(1)			20.00%(1)		40.00%(2)	20.00%(1)				100.00%
5	5	60.00%(3)		20.00%(1)		20.00%(1)						100.00%
6	4	50.00%(2)			25.00%(1)		25.00%(1)					100.00%
7	11	9.09%(1)		9.09%(1)	9.09%(1)	36.36%(4)	9.09%(1)	18.18%(2)			9.09%(1)	100.00%
8	16	62.50%(10)	12.50%(2)	6.25%(1)			6.25%(1)				12.50%(2)	100.00%
9	6	16.67%(1)				33.33%(2)	16.67%(1)	16.67%(1)			16.67%(1)	100.00%
10	11	18.18%(2)	9.09%(1)		9.09%(1)	9.09%(1)	9.09%(1)		18.18%(2)	18.18%(1)	9.09%(1)	100.00%
11	4		25.00%(1)			25.00%(1)	25.00%(1)				25.00%(1)	100.00%
12	3	66.67%(2)			33.33%(1)							100.00%
13	11	45.45%(5)		9.09%(1)	9.09%(1)	9.09%(1)	18.18%(2)				9.09%(1)	100.00%
14	3	66.67%(2)				33.33%(1)						100.00%
15	11	27.27%(3)		18.18%(2)		9.09%(1)	27.27%(3)	9.09%(1)		9.09%(1)		100.00%
16	1	100%(1)										100.00%
17	3	33.33%(1)		33.33%(1)			33.33%(1)					100.00%
18	4				25.00%(1)	50.00%(2)					25.00%(1)	100.00%
19	2							50.00%(1)			50.00%(1)	100.00%
21	8	37.50%(3)				12.50%(1)	25.00%(2)				25.00%(2)	100.00%
22	9		11.11%(1)	33.33%(3)			11.11%(1)	22.22%(2)	11.11%(1)		11.11%(1)	100.00%
23	2					50.00%(1)		50.00%(1)				100.00%
24	5	20.00%(1)		20.00%(1)					20.00%(1)	20.00%(1)	20.00%(1)	100.00%
Grand Total	124	30.65%(38)	4.03%(5)	8.87%(11)	5.65%(7)	12.90%(16)	13.71%(17)	7.26%(9)	3.23%(4)	3.23%(4)	10.48%(13)	100.00%

## References

[1] Fulkerson W.J., McKean K., Nandra K.S., Barchia I.M. (2005). Benefits of accurately allocating feed on a daily basis to dairy cows grazing pasture. Aust. J. Exp. Agric..

[2] Anderson D.M. (2007). Virtual fencing—Past, present and future. Rangel. J..

[3] Anderson D.M., Estell R.E., Holechek J.L., Ivey S., Smith G.B. (2014). Virtual herding for flexible livestock management—A review. Rangel. J..

[4] Umstatter C. (2011). The evolution of virtual fences: A review. Comput. Electron. Agric..

[5] Lee C., Henshall J.M., Wark T.J., Crossman C.C., Reed M.T., Brewer H.G., O’Grady J., Fisher A.D. (2009). Associative learning by cattle to enable effective and ethical virtual fences. Appl. Anim. Behav. Sci..

[6] Lee C., Prayaga K., Reed M., Henshall J. (2007). Methods of training cattle to avoid a location using electrical cues. Appl. Anim. Behav. Sci..

[7] Lomax S., Colusso P., Clark C.E.F. (2019). Does virtual fencing work for grazing dairy cattle?. Animals.

[8] McGreevy P., Boakes R. (2011). Carrots and Sticks: Principles of Animal Training.

[9] Mills D.S. (1998). Applying learning theory to the management of the horse: The difference between getting it right and getting it wrong. Equine Vet. J. Suppl..

[10] Kearton T., Marini D., Cowley F., Belson S., Lee C. (2019). The effect of virtual fencing stimuli on stress responses and behavior in sheep. Animals (Basel).

[11] Campbell D.L.M., Lea J.M., Keshavarzi H., Lee C. (2019). Virtual fencing is comparable to electric tape fencing for cattle behavior and welfare. Front. Vet. Sci..

[12] Domjan M. (2005). Pavlovian conditioning: A functional perspective. Annu. Rev. Psychol..

[13] Lee C., Colditz I.G., Campbell D.L.M. (2018). A framework to assess the impact of new animal management technologies on welfare: A case study of virtual fencing. Front. Vet. Sci..

[14] Gottlieb D.A., Begej E.L., McSweeney F.K., Murphy E.S. (2014). Principles of Pavlovian conditioning: Description, content, function. The Wiley-Blackwell Handbook of Operant and Classical Conditioning.

[15] Lomax S., Colusso P., Gargulio J., Clark C. (2019). Determining learning and behavioural response to a virtual fence for dairy cows. Animals.

[16] Campbell D.L.M., Lea J.M., Farrer W.J., Haynes S.J., Lee C. (2017). Tech-savvy beef cattle? How heifers respond to moving virtual fence lines. Animals.

[17] Campbell D.L.M., Haynes S.J., Lea J.M., Farrer W.J., Lee C. (2019). Temporary exclusion of cattle from a riparian zone using virtual fencing technology. Animals.

[18] Campbell D.L.M., Lea J.M., Haynes S.J., Farrer W.J., Leigh-Lancaster C.J., Lee C. (2018). Virtual fencing of cattle using an automated collar in a feed attractant trial. Appl. Anim. Behav. Sci..

[19] Murphy E.S., Lupfer G.J., McSweeney F.K., Murphy E.S. (2014). Basic principles of operant conditioning. The Wiley-Blackwell Handbook of Operant and Classical Conditioning.

[20] Tucker C.B., Jensen P. (2017). Behaviour of Cattle. The Ethology of Domestic Animals: An Introductory Text.

[21] Olsson A., Seel N.M. (2011). Social learning of fear. Encyclopedia of the Sciences of Learning.

[22] Galef B.G., Laland K.N. (2005). Social learning in animals: Empirical studies and theoretical models. Bioscience.

[23] Gariépy J.-F., Watson K.K., Du E., Xie D.L., Erb J., Amasino D., Platt M.L. (2014). Social learning in humans and other animals. Front. Neurosci..

[24] Mendl M., Nicol C.J., Jensen P. (2017). Learning and Cognition. The Ethology of Domestic Animals: An Introductory Text.

[25] Costa J.H.C., Costa W.G., Weary D.M., Machado Filho L.C.P., Von Keyserlingk M.A.G. (2016). Dairy heifers benefit from the presence of an experienced companion when learning how to graze. J. Dairy Sci..

[26] Bailey D.W., Howery L.D., Boss D.L. (2000). Effects of social facilitation for locating feeding sites by cattle in an eight-arm radial maze. Appl. Anim. Behav. Sci..

[27] Vieira A.D.P., Von Keyserlingk M.A.G., Weary D.M. (2012). Presence of an older weaned companion influences feeding behavior and improves performance of dairy calves before and after weaning from milk. J. Dairy Sci..

[28] Roberts W.A., McSweeney F.K., Murphy E.S. (2014). Animal Cognition. The Wiley-Blackwell Handbook of Operant and Classical Conditioning.

[29] Wredle E., Munksgaard L., Spörndly E. (2006). Training cows to approach the milking unit in response to acoustic signals in an automatic milking system during the grazing season. Appl. Anim. Behav. Sci..

[30] Wredle E., Rushen J., De Passillé A.M., Munksgaard L. (2004). Training cattle to approach a feed source in response to auditory signals. Can. J. Anim. Sci..

[31] Howery L.D., Cibils A.F., Anderson D.M. (2013). Potential for using visual, auditory and olfactory cues to manage foraging behaviour and spatial distribution of rangeland livestock. CAB Rev. Perspect. Agric. Vet. Sci. Nutr. Nat. Resour..

[32] Gutmann A.K., Špinka M., Winckler C. (2020). Do familiar group mates facilitate integration into the milking group after calving in dairy cows?. Appl. Anim. Behav. Sci..

[33] Corbett J.L., Freer M., Graham N.M. (1987). A generalised equation to predict the varying maintenance metabolism of sheep and cattle. Energy Metab. Farm Anim..

[34] Schein M.W., Fohrman M.H. (1955). Social dominance relationships in a herd of dairy cattle. Br. J. Anim. Behav..

[35] Beilharz R.G., Mylrea P.J. (1963). Social position and movement orders of dairy heifers. Anim. Behav..

[36] Bowen D.W., Brooks R.J. (1978). Social organization of confined male collared lemmings ( dicrostonyx groenlandicus traill). Anim. Behav..

[37] Markus S.B., Bailey D.W., Jensen D. (2014). Comparison of electric fence and a simulated fenceless control system on cattle movements. Livest. Sci..

[38] Umstatter C., Morgan-Davies J., Waterhouse T. (2015). Cattle responses to a type of virtual fence. Rangel. Ecol. Manag..

[39] Rstudio T. (2015). Rstudio: Integrated Development for R.

[40] R Core Team (2013). R: A Language and Environment for Statistical Computing.

[41] Bates D., Mächler M., Bolker B., Walker S. (2014). Fitting linear mixed-effects models using lme4. arXiv.

[42] Ripley B.D. (2002). Modern Applied Statisitcs with S.

[43] Hartig F. Dharma: Residual Diagnostics for Hierarchical (Multi-Level/Mixed) Regression Models. https://github.com/florianhartig/DHARMa.

[44] Akaike H. (1974). A new look at the statistical model identification. IEEE Trans. Autom. Control.

[45] Dohoo I., Martin W., Stryhn H.E. (2003). Model-Building Strategies. Veterinary Epidemiologic Research.

[46] Dohoo I., Martin W., Stryhn H.E. (2003). Logistic Regression. Veterinary Epidemiologic Research.

[47] Lenth R., Singmann H., Love J., Buerkner P., Herve M. Emmeans: Estimated Marginal Means, Aka Least-Squares Means. https://github.com/rvlenth/emmeans.

[48] McKillop I.G., Sibly R.M. (1988). Animal behaviour at electric fences and the implications for management. Mamm. Rev..

[49] McDonald C.L., Beilharz R.G., McCutchan J.C. (1981). Training cattle to control by electric fences. Appl. Anim. Ethol..

[50] Wechsler B. (1995). Coping and coping strategies: A behavioural view. Appl. Anim. Behav. Sci..

[51] Quigley T.M., Sanderson H.R., Tiedemann A.R., McInnis M.L. (1990). Livestock control with electrical and audio stimulation. Rangelands.

